# Pro-inflammatory polarization and colorectal cancer modulate alternative and intronic polyadenylation in primary human macrophages

**DOI:** 10.3389/fimmu.2023.1182525

**Published:** 2023-06-08

**Authors:** Joana Wilton, Filipa Lopes de Mendonça, Isabel Pereira-Castro, Michael Tellier, Takayuki Nojima, Angela M. Costa, Jaime Freitas, Shona Murphy, Maria Jose Oliveira, Nicholas J. Proudfoot, Alexandra Moreira

**Affiliations:** ^1^Graduate Program in Areas of Basic and Applied Biology (GABBA) PhD Program, ICBAS-Instituto de Ciências Biomédicas Abel Salazar, Universidade do Porto, Porto, Portugal; ^2^Gene Regulation - Instituto de Investigação e Inovação em Saúde, Universidade do Porto, Porto, Portugal; ^3^IBMC-Instituto de Biologia Molecular e Celular Universidade do Porto, Porto, Portugal; ^4^Sir William Dunn School of Pathology, University of Oxford, Oxford, United Kingdom; ^5^Tumour and Microenvironment Interactions Group – Instituto de Investigação e Inovação em Saúde, Universidade do Porto, Porto, Portugal; ^6^INEB-Instituto Nacional de Engenharia Biomédica Universidade do Porto, Porto, Portugal; ^7^Faculdade de Medicina, Universidade do Porto, Porto, Portugal; ^8^ICBAS- Instituto de Ciências Biomédicas Abel Salazar, Universidade do Porto, Porto, Portugal

**Keywords:** primary human macrophages, mRNA, alternative polyadenylation, cancer, RNA sequencing, intronic polyadenylation; 3'UTR; SRSF12

## Abstract

**Introduction:**

Macrophages are essential cells of the immune system that alter their inflammatory profile depending on their microenvironment. Alternative polyadenylation in the 3’UTR (3’UTR-APA) and intronic polyadenylation (IPA) are mechanisms that modulate gene expression, particularly in cancer and activated immune cells. Yet, how polarization and colorectal cancer (CRC) cells affect 3’UTR-APA and IPA in primary human macrophages was unclear.

**Methods:**

In this study, we isolated primary human monocytes from healthy donors, differentiated and polarized them into a pro-inflammatory state and performed indirect co-cultures with CRC cells. ChrRNA-Seq and 3’RNA-Seq was performed to quantify gene expression and characterize new 3’UTR-APA and IPA mRNA isoforms.

**Results:**

Our results show that polarization of human macrophages from naïve to a pro-inflammatory state causes a marked increase of proximal polyA site selection in the 3’UTR and IPA events in genes relevant to macrophage functions. Additionally, we found a negative correlation between differential gene expression and IPA during pro-inflammatory polarization of primary human macrophages. As macrophages are abundant immune cells in the CRC microenvironment that either promote or abrogate cancer progression, we investigated how indirect exposure to CRC cells affects macrophage gene expression and 3’UTR-APA and IPA events. Co-culture with CRC cells alters the inflammatory phenotype of macrophages, increases the expression of pro-tumoral genes and induces 3’UTR-APA alterations. Notably, some of these gene expression differences were also found in tumor-associated macrophages of CRC patients, indicating that they are physiologically relevant. Upon macrophage pro-inflammatory polarization, *SRSF12* is the pre-mRNA processing gene that is most upregulated. After *SRSF12* knockdown in M1 macrophages there is a global downregulation of gene expression, in particular in genes involved in gene expression regulation and in immune responses.

**Discussion:**

Our results reveal new 3’UTR-APA and IPA mRNA isoforms produced during pro-inflammatory polarization of primary human macrophages and CRC co-culture that may be used in the future as diagnostic or therapeutic tools. Furthermore, our results highlight a function for *SRSF12* in pro-inflammatory macrophages, key cells in the tumor response.

## Introduction

Communication between neighboring cell types induces alterations in gene expression. In colorectal cancer (CRC), one of the deadliest forms of cancer, the recruited immune cells are influenced by the tumor heterogeneity and by their microenvironment (1). Macrophages are abundant innate immune cells in the tumor microenvironment, that either cooperate with or abrogate cancer progression, depending on their inflammatory profile (2, 3) and are involved in immune evasion, which is a hallmark of tumorigenic progression (4). Importantly, they show transcriptional programs that control pro- and anti-inflammatory cellular responses, enabling a spectrum of phenotypes to respond to each stimulus (5). Naïve macrophages (hereby referred to as M0) may be polarized in a continuum of inflammatory populations, ranging from pro-inflammatory (M1-like, hereby referred to as M1) to anti-inflammatory (M2-like, hereby referred to as M2). At the onset of CRC progression, M1 macrophages recognize and attack tumor cells (6, 7). Yet, the cancer cells that escape, acquire increased invasive and metastatic properties, creating a tumor-permissive microenvironment, within which macrophages develop a profile similar to M2 (3, 8).

Transcriptomic studies have highlighted the number and biological relevance of mRNA isoforms produced by alternative polyadenylation in the 3’UTR (3’UTR-APA) and by intronic polyadenylation (IPA) (9). Both APA and IPA contribute to gene expression regulatory mechanisms (10–12), with consequences for cell proliferation, differentiation, and cell cycle (13–17). APA occurs in more than 70% of human genes (18–21), and determines mRNA half-life and subcellular location, as well as protein subcellular localization and function (22, 23). IPA occurs due to the recognition of polyadenylation signals (PASs) within introns, producing truncated transcripts that either are non-functional or code for proteins lacking the C-terminus (24). IPA has been described in immune cells (25), in leukemia (26) and occurs in physiological relevant genes such as the gene for the core cleavage and polyadenylation protein *PCF11* (27). In cancer and activated T cells, mRNAs with short 3’UTRs due to 3’UTR-APA are more prevalent than those with long 3’ UTRs (12, 28–30). It has also been shown that shorter IPA mRNA isoforms contribute to increased transcriptomic diversity in primary multiple myeloma cells and in naïve B cells, memory B cells, germinal center B cells, CD5^+^ B cells, T cells and plasma cells (25), but the prevalence of IPA events in primary human macrophages has not been reported so far.

Here, we address how polarization of primary human macrophages and co-culture with CRC cells affect fundamental processes such as gene expression, 3’UTR-APA and IPA, in order to identify transcriptomic alterations in relevant mRNA isoforms, that we term signatures. We isolated primary human monocytes from healthy donors, exposed them to pro-inflammatory conditions and subsequently co-cultured them with two different CRC cell lines. By 3’RNA-Seq, we identified a robust 35-gene signature of differentially-expressed genes (DEGs) in M1 polarized macrophages, comprising of inflammatory and immune-related genes. Noteworthy, some of these gene expression alterations are observed in tumor-associated macrophages in CRC patients. In addition, M1 polarization of primary human macrophages induces proximal PAS usage, leading to the production of mRNAs with short 3’UTRs in physiologically relevant genes, such as *IL17RA* and *TP53RK*. IPA events are increased in M1 polarized macrophages affecting genes such as *MAP3K8* and *WDR33*. Both 3’UTR-APA shortening and IPA are further upregulated after CRC co-culture, in particular for *MAP3K8* and *WDR33*. *SRSF12* expression is strongly upregulated in primary human macrophages upon M1 polarization. Notably, *SRSF12* knockdown causes a strong downregulation in the expression of genes involved in regulation of gene expression and in macrophage functions. Moreover, *SRSF12* knockdown leads to an increase in *MBNL1* proximal PAS selection and an increase in *CDC42* IPA. Our results describe 3’UTR-APA and IPA events in primary human macrophages driven by M1 polarization and by exposure to colorectal cancer cells, which are relevant for gene expression regulation and macrophage functions. Furthermore, our results show a new function for *SRSF12* in pro-inflammatory macrophages, which may be important for the tumor response.

## Results

### Pro-inflammatory polarization alters primary human macrophages transcription profile

**Figure 1:**
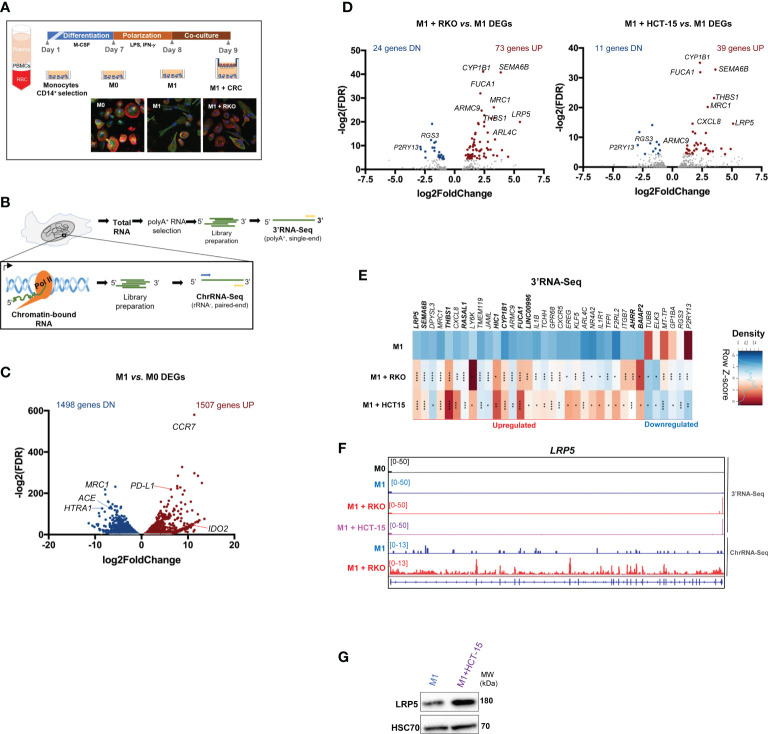
M1 polarization and CRC co-culture modulate macrophage inflammatory and gene expression profiles **(A)** Top, experimental setup: PBMCs are isolated through gradient centrifugation and then exposed to CD14^+^ beads to select CD14^+^ monocytes. Monocytes are differentiated for 7 days to obtain unpolarized naïve M0 macrophages and then polarized to M1-like (M1) macrophages for 24 hours. M1 macrophages are co-cultured with CRC cell lines RKO or HCT15 for 24h. Bottom, confocal microscopy images show actin-tubulin cytoskeleton of (M0), LPS+IFN-γ stimulated pro-inflammatory (M1) polarized macrophages, and LPS+IFN-γ-stimulated macrophages co-cultured with CRC cell line (M1 + RKO). DNA stained in blue, actin stained in red, tubulin stained in green. **(B)** Setup of extraction and library preparation for RNA-Seq of primary human macrophages. rRNA-depleted chromatin-bound RNA were converted into paired-read libraries for ChrRNA-Seq, and polyA^+^-enriched total RNA were converted into single-end libraries for 3’RNA-Seq. **(C)** Volcano plot of 3’RNA-Seq data comparing differentially-expressed genes (DEGs) in M1 *vs*. M0. Blue and red dots represent statistically significant genes with log2(Fold Change) <-1 or >1, respectively. **(D)** Volcano plot of 3’RNA-Seq data comparing differentially-expressed genes (DEGs) in M1 macrophages co-cultured with RKO cells (M1+ RKO, left) or HCT-15 cells (M1 + HCT15, right) *vs*. M1 macrophages in monocultures (M1). Red dots represent statistically significant upregulated genes (log2(Fold Change >1); blue dots represent statistically significant downregulated genes (log2(Fold Change <-1). Gray dots represent non-significantly expressed genes. **(E)** Representative donor heatmap of 3’RNA-Seq DEGs in macrophages co-cultured with RKO (M1 + RKO) or HCT15 (M1 + HCT15) *vs*. M1 macrophages in monocultures, ordered by fold change. n=3 healthy donors, p-value calculated using DESeq2 test through the Benjamini-Hochberg method: *p< 0,05; **p <0,01; ***p< 0,005; ****p<0,0001. Genes in bold in the figure correspond to the DEGs occurring in both 3’RNA-Seq and ChrRNA-Seq datasets. **(F)** *LRP5* representative gene profile and transcript counts in 3’RNA-Seq (top) and ChrRNA-Seq (log2FoldChange= 5.777401; pvalue= 1.50E-24 and padj= 2.98E-20) (bottom). M0 macrophages (black), M1 macrophages cultured alone (blue) and M1 macrophages co-cultured with the CRC cell lines RKO (red) or HCT-15 (lilac) visualized in IGV **(G)** Western blot showing that LRP5 protein levels also increase upon CRC co-culture.

Macrophages can be experimentally differentiated from quasi-naïve, differentiated and unpolarized cells into a spectrum of inflammatory profiles. We obtained primary human CD14^+^ monocytes isolated from peripheral blood mononuclear cells (PBMCs) of healthy blood donor buffy coats, differentiated them into macrophages in the presence of M-CSF (herein referred as M0) and polarized them toward pro-inflammatory conditions (M1) by incubation with LPS and IFN-γ, following a well-established methodology (31–33) represented in **Figure 1A**. M0 macrophages are predominantly round/ameboid-like, while M1 macrophages present a fibroblast-like morphology (**Figure 1A**). Characterization of the macrophage inflammatory profile shows that upon M1 polarization the CD14 monocytic cell lineage marker is maintained (**Figure S1A**), the expression of CD86, CCR7 and IL1β pro-inflammatory markers is increased, and the expression of CD163 and TGF-β anti-inflammatory markers is decreased (**Figures S1B–E**), thus showing that M1-polarized macrophages present a *bona fide* pro-inflammatory phenotype.

To assess alterations in gene expression, 3’UTR-APA and IPA events upon macrophage polarization, we performed 3’RNA-Seq, which provides both quantification of gene expression based on RNA levels at the 3’ end and APA profiles (32) and chromatin-bound RNA-Seq (ChrRNA-Seq) to pinpoint those events that occur in nascent RNA (34) (**Figure 1B**). 3’RNA-Seq reveals that 3005 genes have significantly altered expression in M1 in comparison to M0 macrophages (**Figure 1C**). The upregulated 1507 gene subset contains genes involved in stress and immune responses, IFN-γ and TNF-α signaling pathways, all of which are characteristic of pro-inflammatory responses (**Figure S1F**). Of note, the pro-inflammatory markers *CCR7*, *CCL19*, *IDO2*, *IL7R* and the *PD-L1* immune checkpoint are present among the upregulated gene subset (**Figure 1C**; **Supplementary Data 1**). Conversely, the downregulated 1498 gene expression subset (**Figure 1C**; **Supplementary Data 1**) was significantly enriched in GSEA terms associated with extracellular matrix (ECM), secretory vesicles and endocytosis, lipid binding, and GPCR signaling (**Figure S1F**), which are classically associated with anti-inflammatory macrophages (35). These results indicate that M1 polarization leads to a phenotypically and transcriptomically robust pro-inflammatory profile.

### CRC co-culture induces a 35-gene expression signature in primary human macrophages

At the early stages of CRC progression, tumor-associated macrophages (TAMs) are described to harbor a more M1-like tumor-inhibitory phenotype, which upon CRC exposure may become an M2-like anti-inflammatory state (6, 7). Therefore, we polarized macrophages into M1 and co-cultured them with the CRC cell lines RKO and HCT-15 (36) to analyze the impact of exposure to cancer cells on the macrophage transcriptome (**Figure 1A**). Both CRC cell lines belong to the CMS1 (consensus molecular subtype 1), characterized by hypermutation, high microsatellite instability and pronounced immunogenicity (37), but differ in several tumorigenic markers such as APC, K-ras, B-raf, TGFBR2 and MLH1 (38). By using these two CRC cell lines we can determine which effects are specific to each cell line, and which are general features of CRC exposure. Upon co-culture with CRC cells, macrophage morphology reverts to an ameboid-like phenotype, similar to M0, a phenotypic alteration constant in all replicates and with both CRC cell lines (**Figures 1A**, **S2A**), while still maintaining the CD14 and CD86 markers (**Figures S2B, C**). 3’RNA-Seq analyses of the macrophages revealed that RKO and HCT-15 co-culture induce alterations in gene expression (**Figure 1D**; **Supplementary Data 1**). The 3’RNA-Seq Pearson correlation analysis indicate a very high correlation between biological replicates (**Figure S2D**) and a PCA analysis shows that samples also group by polarization state (**Figure S2E**).

We identified 35 differentially-expressed genes (DEGs) in macrophages that are common to co-culture with both CRC cell lines, including 29 upregulated and 6 downregulated genes (**Figures 1E**). The 35 DEGs signature includes 15 anti-inflammatory and/or pro-tumorigenic genes, including *LRP5, SEMA6B*, *MRC1, ARMC9, FUCA1, EREG* and *ARL4C*, genes related to immune modulation and inflammation, such as *THBS1, CXCL8*, *LY6K* and *KLF5*, and pro-inflammatory genes, such as *IL1B, CXCR5, IL1R1* and *F2RL2*. Some of these DEGs were validated by RT-qPCR (**Figure S2F**) in sequenced and non-sequenced samples. These genes were selected based on their function in macrophage biology, in particular by their inflammatory profile in the tumor niche. Genes showing upregulation of expression (*SEMA6B* and *THBS1*) were selected due to their presence in both 3’RNA-Seq and ChrRNA-Seq datasets. Other anti-inflammatory and/or pro-tumoral significantly upregulated DEGs in macrophages co-cultured with CRC cells include *SERPINB2*, *MS4A6A*, *TGFA*, *FOSB*, *FABP4* and *RGS1* (**Figure S2G**). These results indicate that macrophages change their inflammatory profile upon CRC co-culture.

We then asked how the changes observed upon co-culture with CRC cells correlate with changes that occur during M0 to M1 polarization. As it can be observed in the Venn analysis of **Figure S2H**, there are only 19 common genes to both conditions and from these only 6 are upregulated in all datasets: *CXCR5, EREG, F2RL2, ITGB7, KLF5, TCHH* (39–41).

### The 35-gene expression signature is also present in macrophages of CRC patients

Some of the most upregulated genes found in CRC co-cultured macrophages – *LRP5, DPYSL3*, *THBS1*, *CXCL8*, *IL1ß* and *EREG* (**Figure 1E**) – are related to the Wnt pathway (42–46), which is constitutively activated in CRC (47). *LRP5* is poorly expressed in M1 macrophages, but it is induced upon co-culture with CRC as shown by the Volcano plot (**Figure 1D**), by the heatmap (**Figure 1E**), by the Integrative Genomics Viewer (IGV) of the 3’RNA-Seq and Chromatin bound RNA-Seq (ChrRNA-Seq) data (**Figure 1F**), and by the increase in LRP5 protein levels (**Figure 1G**). ChrRNA-Seq allows investigation of the nascent RNA that is still bound to the chromatin. Comparing the RNA profiles obtained by 3’RNA-Seq and ChrRNA-Seq as shown for LRP5 (**Figure 1F**), indicates that the changes in gene expression observed are not due to post-transcriptional regulation.

**Figure 2:**
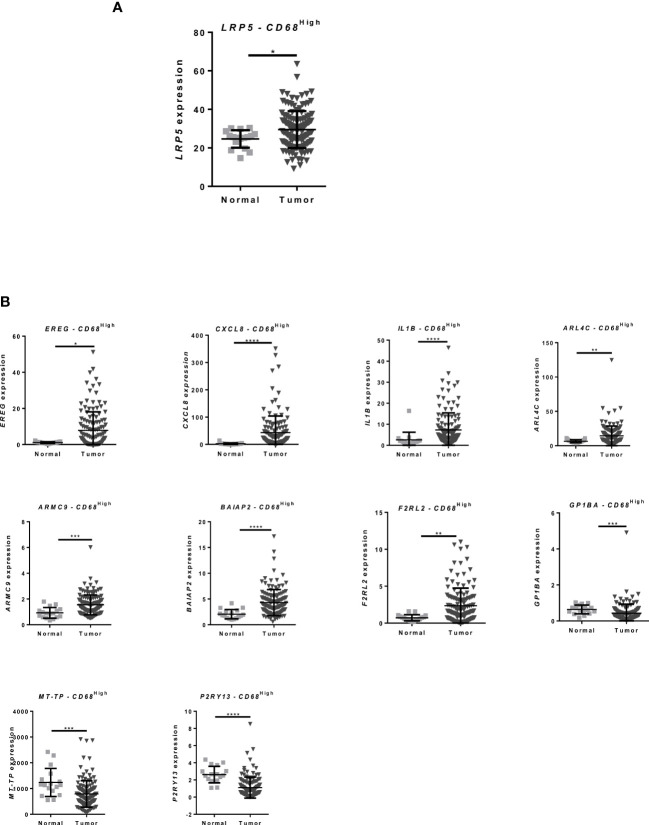
CRC patients present differential expression of inflammation-related genes that is also found in CRC co-cultured macrophages **(A)** Expression data from CRC patients from the TCGA database: Microarray and RNA-Seq gene expression of *LRP5* in CD68^High^ macrophage population between normal and tumor tissue; **(B)** *EREG*, *CXCL8*, *IL1B ARL4C*, *ARMC9*, *BAIAP2*, *F2RL2*, *GP1BA*, *MT-TP* and *P2RY13* expression in CRC patients in CD68^High^ macrophage population between normal and tumor tissue, data from TCGA database Mann–Whitney test was used to compare gene expression between groups. **** p < 0.0001; *** p < 0.001; ** p < 0.01; * p < 0.05.

To evaluate whether this upregulation occurs in clinically relevant contexts, we analyzed gene expression levels in CRC patients, using RNA-Seq data from The Cancer Genome Atlas (TCGA) database and selecting CD68^High^ tumor associated macrophages (TAMs). Notably, *LRP5* is upregulated in macrophages from CRC patients (**Figure 2A**). Furthermore, several other genes in the 35 gene DGE signature presented by macrophages after co-culture with CRC (from **Figure 1E**), including *EREG*, *CXCL8*, *IL1B*, *ARL4C*, *ARMC9*, *BAIAP2*, *F2RL2*, *GP1BA*, *MT-TP* and *P2RY13*, show the same expression profile in TAMs from CRC patients (**Figure 2B** and **Supplementary Figure S3**). These results indicate that CRC co-culture induces the expression of genes that are similarly expressed by TAMs from CRC patients.

### Pro-inflammatory polarization induces 3’UTR-APA shortening and IPA in primary human macrophages

To investigate the effect of M1 polarization in 3’UTR-APA and IPA we analyzed the 3’RNA-Seq data of M1 *vs*. M0 macrophages, using APAlyzer (48) and obtained a dataset consisting of 3'UTR-APA and IPA mRNA isoforms relative expression differences (**Supplementary Data 2**).

**Figure 3:**
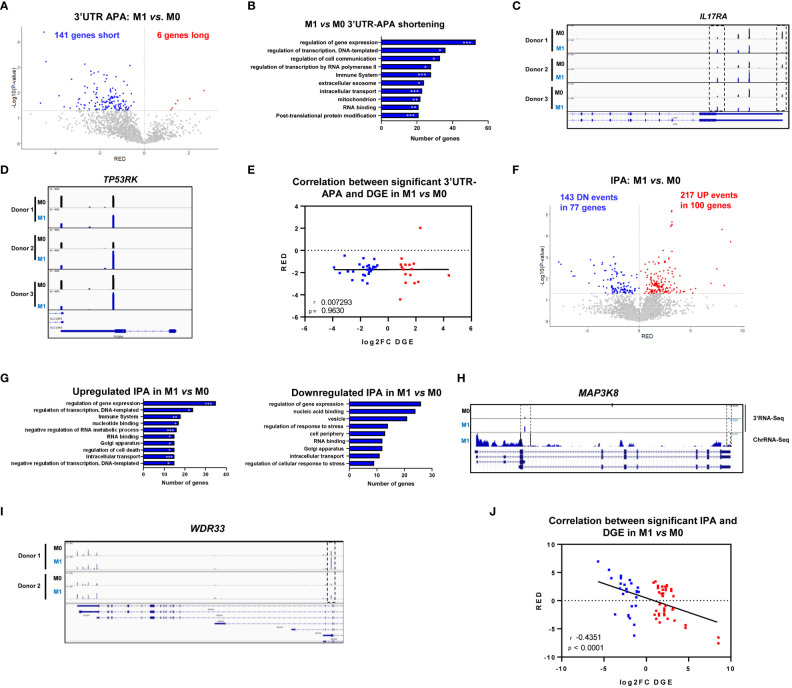
M1 polarization induces 3’UTR-APA shortening and intronic polyadenylation (IPA). **(A)** Volcano plot of genes showing differential 3’UTR-APA mRNA isoforms in M1 *vs*. M0. Red dots represent genes with Relative Expression Differences (RED)>0, corresponding to expression of the long isoform, and blue dots represent genes with RED<0, corresponding to expression of the short isoform and p<0.05, gray dots represent genes without significantly different RED. **(B)** Top 10 Gene Ontology terms in M1 *vs*. M0 of genes showing upregulated short 3’UTR. Gene Ontology p-value was calculated using Fisher’s test (Panther). *p<0,05; **p<0,01; ***p<0,005. **(C)** *IL17RA* gene profile and transcript counts in 3’RNA-Seq visualized in IGV for 3 different healthy donors. M0 macrophages (black), M1 macrophages cultured alone (blue) visualized in IGV. Dashed boxes indicate the reads correspondent to short (proximal PAS usage) and long (distal PAS usage) 3’UTRs mRNA isoforms. **(D)** *TP53RK* gene profile and transcript counts in 3’RNA-Seq visualized in IGV for 3 different healthy donors. M0 macrophages (black), M1 macrophages cultured alone (blue) visualized in IGV. **(E)** Two-tailed Pearson correlation between significant differential 3’UTR-APA genes (RED) and DGE (log2FC DGE) in M1 *vs* M0 macrophages (n=3 donors). Genes shown in blue have significant downregulation of expression, genes shown in red have significant upregulation of expression. **(F)** Volcano plot of genes showing differential IPA mRNA isoforms in M1 *vs*. M0 analyzed by Apalyzer (genes that undergo an increase in IPA events are represented in red (RED>0), UP: upregulated IPA; genes that undergo a decrease in IPA events, hence upregulating expression of last exon are represented in blue, DN: downregulated IPA, RED < 0) and p < 0.05. **(G)** Top 10 Gene Ontology terms in M1 *vs*. M0 of genes showing differential IPA. Gene Ontology p-value calculated using Fisher’s test (Panther). *p<0,05; **p<0,01; ***p<0,005. **(H)** Gene profiles and transcript counts of *MAP3K8* obtained through 3’-RNA-Seq (top) and ChrRNA-Seq (down) of M0 macrophages (black) and M1 macrophages (blue), visualized in IGV. **(I)** IGV gene profiles and transcript counts of *WDR33* obtained through 3’RNA-Seq of M0 macrophages (black) and M1 macrophages (blue) in two different donors. Dashed box indicates the reads correspondent to the IPA mRNA isoform. **(J)** Two-tailed Pearson correlation between significant differential IPA (RED) and DGE (log2FC DGE) in M1 *vs* M0 macrophages (n=3 donors). Genes shown in blue have significant downregulation of expression, genes shown in red have significant upregulation of expression.

We observed a strong upregulation of mRNA isoforms with short 3’UTRs resulting from proximal PAS selection as 141 genes showed upregulation of short 3’UTRs compared to 6 genes showing upregulation of long 3’UTRs (**Figure 3A**). Genes that undergo 3’UTR shortening showed GO terms involved in regulation of gene expression, and also related to the immune system, cell-cell communication, extracellular exosomes and intracellular transport (**Figure 3B**), which are important for macrophage inflammatory functions. *IL17RA* (Interleukin 17 receptor A) is an example of a gene with upregulation of the shortest 3’UTR mRNA isoform and downregulation of the longest 3’UTR in M1 *vs*. M0 (**Figure 3C**). Interleukin 17A and its receptor IL17RA play a pathogenic role in many inflammatory and autoimmune diseases and IL17RA contributes to the inflammatory response by inducing recruitment of innate immune cells (49). In addition, IL17RA deletion predicts high-grade colorectal cancer and poor clinical outcomes (50). *TP53RK* (TP53 Regulating Kinase) is another example of a gene that displays a decrease in the expression of the longest 3’UTR mRNA isoform and an upregulation of the shortest 3’UTR mRNA isoform in M1 *vs* M0 (**Figure 3D**). TP53RK/PRPK enables p53 binding and the expression levels of phosphorylated TP53RK/PRPK are higher in metastatic CRC tissues in comparison to normal tissues (51). Other physiologically relevant genes with upregulated short 3’UTRs upon M1 polarization include *YKT6*, which is connected to the Wnt pathway, lysosome fusion, and exosome secretion (52), *ALYREF*, an RBP (RNA binding protein) that is recruited to target mRNAs through interaction with IRAK2 and binds 5’ and 3’ UTRs through a complex with CSTF2/CstF64 (53, 54) and *MYO10*, whose 3’UTR is regulated by TGFβ signaling *via* SMAD transcription factors (55) (**Supplementary Data 2**). Of note, the 3’UTR-APA changes induced by M1 polarization do not present a significant correlation with differential gene expression, as shown by the two-tailed Pearson correlation between significant genes with both DGE and isoform RED (Relative Expression Differences) in differential APA in M1 *vs* M0 (**Figure 3E**).

When we analyzed IPA, we observed a strong increase in the expression of IPA mRNA isoforms in M1 *vs* M0, as 100 genes showed upregulation of IPA mRNA isoforms, in a total of 217 upregulated IPA events (**Figure 3F**). Upregulation of IPA isoforms showed GO terms connected to regulation of gene expression and the immune system (**Figure 3G**), similarly to the GO terms found for 3’UTR-APA events, while downregulation of IPA mRNA isoforms present GO terms that curiously do not include the immune system, but include regulation of response to stress (**Figure 3G**). Genes that undergo IPA upregulation upon M1 polarization include *MAP3K8*, which is widely expressed in immune cells and tumors (56). In M0, *MAP3K8* uses two PAS, one at the 3’UTR and another PAS in an intron. In M1 the intronic PAS is selected ~3-fold more efficiently than in M0 (**Figure 3H**). This shorter transcript is described in ENSEMBL and encodes a 132 aa, 15kDa MAP3K8 truncated protein isoform predicted by the Uniprot database, Q5T854. It remains to be investigated whether the IPA mRNA isoform we observe corresponds to that protein. *WDR33*, which codes for the protein that binds to the PAS (57), is another gene that undergoes a >2-fold increase in an IPA mRNA isoform during pro-inflammatory polarization (dashed box in **Figure 3I**). This IPA isoform, if functional, produces a shorter WDR33 protein of still unknown function.

Interestingly, we found a strong negative correlation between IPA and gene expression as shown by the two-tailed Pearson correlation between significant genes with both DGE and isoform RED in differential IPA in M1 *vs* M0 macrophages (**Figure 3J**). Genes with an increase in IPA tend to be downregulated, and genes with a decrease in IPA tend to be upregulated.

### CRC co-culture affects 3’UTR-APA and IPA in primary human macrophages

We next analyzed how co-culture with CRC cells affects 3’UTR-APA and IPA in primary human macrophage by analyzing 3’RNA-Seq data with APAlyzer (**Supplementary Data 2**).

**Figure 4:**
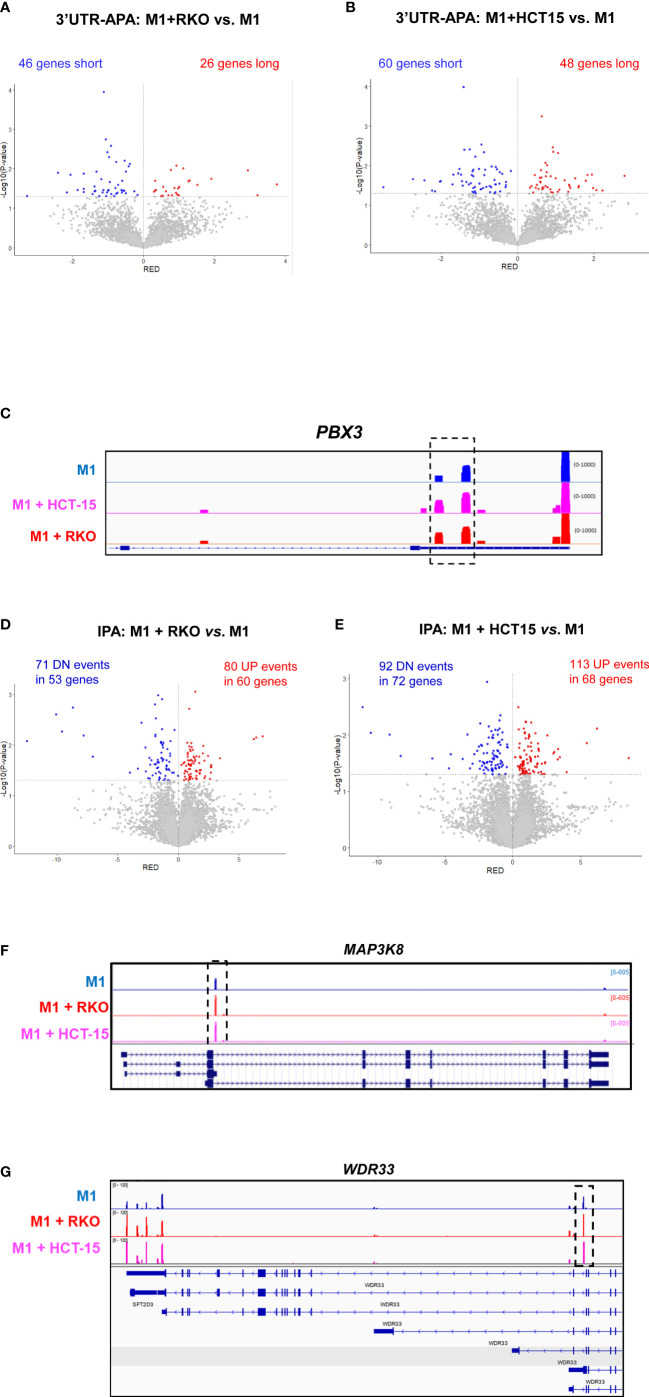
CRC co-culture induces 3’UTR-APA and IPA changes in macrophages. **(A, B).** Volcano plots of genes showing differential 3’UTR-APA changes in M1+RKO *vs*. M1 **(A)** and in M1+HCT15 *vs*. M1 **(B)**. Genes that express short 3’UTRs mRNAs are represented in blue and genes that express long 3’UTRs mRNAs are represented in red. **(C)** IGV gene profile and transcript counts of *PBX3* obtained through 3’RNA-Seq of M1 macrophages (blue) and M1 macrophages co-cultured with the CRC cell lines HCT-15 (lilac) and RKO (red). Dashed box indicates an increase in reads in proximal polyA sites, in CRC co-cultured macrophages. **(D, E)**. Volcano plots of genes showing IPA changes in M1+RKO *vs*. M1 **(D)** and in M1+HCT15 *vs*. M1 **(E)**. Genes that express more IPA mRNAs are represented in red (UP) and genes that express less IPA mRNAs are represented in blue (DN). **(F)** IGV gene profile and transcript counts of *MAP3K8* obtained by 3’RNA-Seq of M1 macrophages (blue) and M1 macrophages co-cultured with the CRC cell lines RKO (red) and HCT-15 (lilac). Dashed box indicates an increase in IPA reads in CRC co-cultured macrophages. **(G)** IGV gene profile and transcript counts of *WDR33* obtained by 3’RNA-Seq of M1 macrophages (blue) and M1 macrophages co-cultured with the CRC cell lines RKO (red) and HCT-15 (lilac). Dashed box indicates an increase in IPA reads in CRC co-cultured macrophages.

Co-culture of M1 primary human macrophages with both RKO and HCT-15 CRC cell lines induces 3’UTR-APA changes, with an increase in the expression of mRNA isoforms with short 3’UTRs (**Figures 4A, B**). The list of genes with differential 3’UTR-APA that are common to both co-cultured macrophages datasets are listed in **Table 1**. Interestingly, common upregulated long 3’UTR genes include *C11orf54* and *STARD3NL*, which are involved in exosome functions (58, 59) and *TMEM265*, whose expression is negatively correlated with tumor-infiltrating immune cells (60), while upregulated short 3’UTR genes include *PBX3* (**Figure 4C**), which is associated with inflammation and promotes migration and invasion of colorectal cancer cells (61–63).

**Table 1:** Differential 3’UTR-APA genes in M1+CRC *vs* M1.

Short 3’UTR	Long 3’UTR
*GGH*	*C11orf54*
*KDM7A*	*DHDDS*
*LMAN1*	*NDOR1*
*PBX3*	*STARD3NL*
*RBMX*	*TIMM9*
*SPCS2*	*TMEM265*

Alphabetical lists of genes showing differential 3’UTR-APA mRNA isoforms that are common to the two macrophage datasets correspondent to the co-cultures with RKO and HCT-15 cell lines.

We also found that, after co-culture with CRC cells, macrophages display an increase in IPA (**Figures 4D, E**). The genes that are common to both co-cultured macrophages datasets in the IPA analysis include 17 genes with upregulated IPA (**Table 2**). This list contains physiologically relevant genes for macrophage and cancer biology, such as *MAP3K8* (**Figure 4F**). We had initially observed that pro-inflammatory polarization caused a ~2-fold increase in a *MAP3K8* IPA mRNA isoform (**Figure 3H**). Notably, co-culture with CRC cells further increases by ~2-fold the expression of this IPA mRNA isoform (**Figure 4F**). *WDR33*, is another interesting example of a gene that expresses an IPA isoform which increases by pro-inflammatory polarization (**Figure 3I**) and that is further increased in macrophages after co-culture with CRC cells (**Figure 4G**). Curiously, the bioinformatic analysis included this gene in the downregulated IPA common list of genes (**Table 2**), as the 3’UTR-APA long mRNA isoform increases. Other genes with upregulated IPA are *CUX1*, which inhibits NF-kB transcriptional activity and contributes toward tumor progression and is a target of TGFß (64) and *MESD*, a chaperone necessary for LRP5 translocation, which is related to the Wnt pathway (65, 66) and *TIA1*, a RBP expressed in activated macrophages and in CRC (67, 68).

**Table 2:** Differential IPA genes in M1+CRC *vs* M1.

Upregulated IPA	Downregulated IPA
*CUX1*	*ACYP2*
*EIF2A*	*ATP6V0A1*
*EIF4G3*	*C15orf38*
*ETNK1*	*CHPF*
*GRK3*	*COX17*
*JDP2*	*FAM91A1*
*MAP3K8*	*MAP3K20*
*MESD*	*NOL4L*
*NRF1*	*PTGS1*
*PLEKHA6*	*RAB6A*
*SIPA1L*	*RERE*
*SMG6*	*SLC9B2*
*TIA1*	*SOGA1*
*THOC2*	*TBC1D5*
*TMED7*	*WDR33*
*WDR20*	*AP3S2*
*TICAM2*	

Alphabetical lists of genes showing upregulated and downregulated IPA that are common to the two macrophage datasets correspondent to the co-cultures with RKO and HCT-15 cell lines.

The fifteen genes common to both co-cultured macrophages datasets that show downregulated IPA also have a physiological function in macrophage and cancer biology (**Table 2**). This list include *COX17*, which provide essential copper transport for M1 macrophages in tumors (69), *RAB6A*, associated with Golgi regulation of TNF secretion in macrophages (70) and *SLC9B2*, which is associated with infiltrating macrophages in CRC (71).

Curiously, some of the genes in the 3'UTR-APA genes in the APA and IPA lists are connected to the Wnt pathway and most of them are related to lipids metabolism, through functions related to the Golgi, ER, mitochondria, or plasma membranes, and exosomes.

Our results show that polarization of primary human macrophages leads to an increase in the expression of 3’UTR-APA short mRNA isoforms and in IPA mRNA isoforms, in physiologically relevant genes. Moreover, CRC co-culture induces even further 3'UTR-APA and IPA changes in macrophages.

### *SRSF12* knockdown decreases gene expression in M1 macrophages

**Figure 5:**
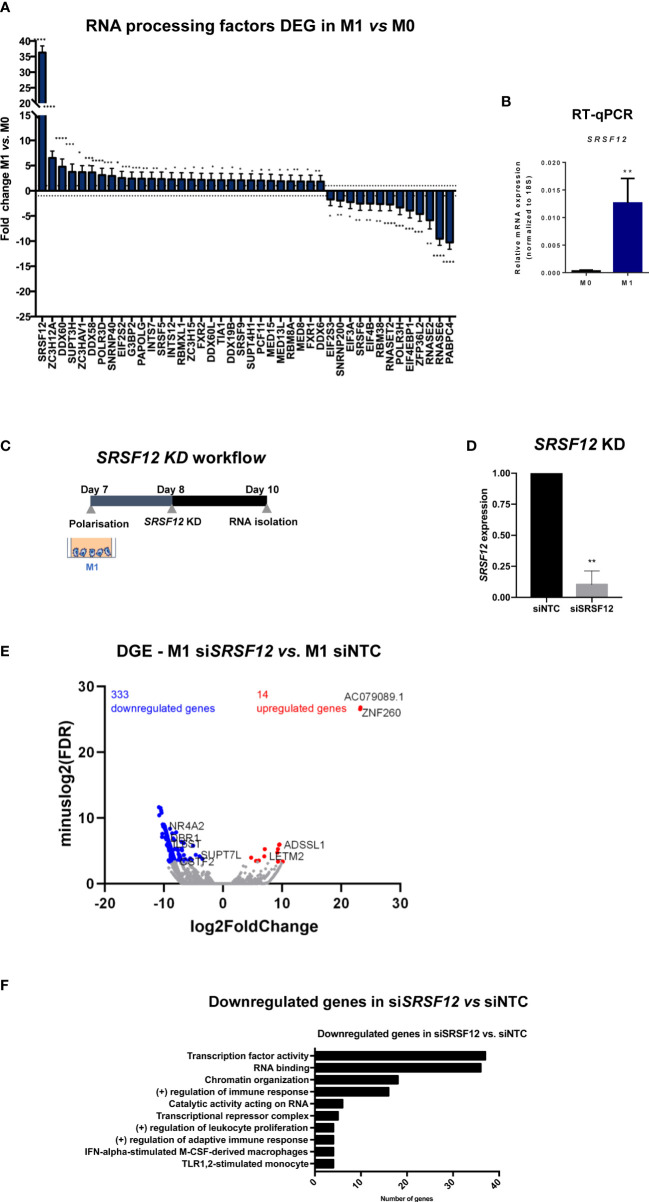
SRSF12 modulates gene expression in pro-inflammatory macrophages. **(A)** Fold change distribution of CPA and transcription regulation DEGs in M1 vs. M0, ordered by fold change. n=3 healthy donors, p-value calculated using DESeq2 test through the Benjamini-Hochberg method: *p< 0,05; **p <0,01; ***p< 0,005; ****p<0,0001. **(B)** *SRSF12* mRNA levels in M1 *vs* M0 quantified by RT-qPCR. n=4 donors, Student’s t-test, p = 0.0202. **(C)** Experimental setup of *SRSF12* knockdown in primary human macrophages. **(D)** Levels of *SRSF12* knockdown quantified by RT-qPCR. n=3 donors, Student’s t-test, p = 0.0050 **(E)** Volcano plot of DEGs in si*SRSF12 vs* siNTC in M1 macrophages in n=2 donors. Red dots represent genes with log2(Fold Change) >1, blue dots genes with log2(Fold Change) <– 1 and p < 0.1, gray dots represent non-significantly expressed genes. **(F)** GSEA terms of downregulated DEGs in si*SRSF12 vs* siNTC. n= 2, p-value calculated using GSEA: *p < 0,05; **p < 0,01; ***p < 0,005.

To understand the mechanisms behind the transcriptomic alterations observed following macrophage M1 polarization, we analyzed the 3’RNA-Seq data to identify changes in the expression of genes that code for proteins involved in cleavage and polyadenylation, splicing and transcription termination (**Figure 5A**). Of these genes, the most upregulated gene in M1 macrophages is the splicing repressor *SRSF12/SRrp35* (72) (**Figures 5A, B**). Therefore, we knocked down *SRSF12* in primary macrophages after M1 polarization using siRNAs (**Figure 5C**) and achieved >80% of *SRSF12* mRNA depletion (**Figure 5D**). Interestingly, *SRSF12*-depleted macrophages show a marked downregulation of gene expression (333 downregulated genes *vs.* 14 upregulated genes) (**Figure 5E**; **Supplementary Data 3**). Notably, several genes involved in gene expression regulation are downregulated, in particular *CSTF2/CstF64, SRSF2*, *SRSF3*, *U2SURP*, *DBR1*, *PARP2*, *EIF5A2*, as well as several transcription factors such as *NFYA*, *EGR1*, *ELK3*, *SMAD1* and *MTA3* (**Figure 5F**; **Supplementary Data 3**). Regulation of immune responses and leukocyte proliferation are functions also present in downregulated genes (**Figure 5F**).

**Figure 6:**
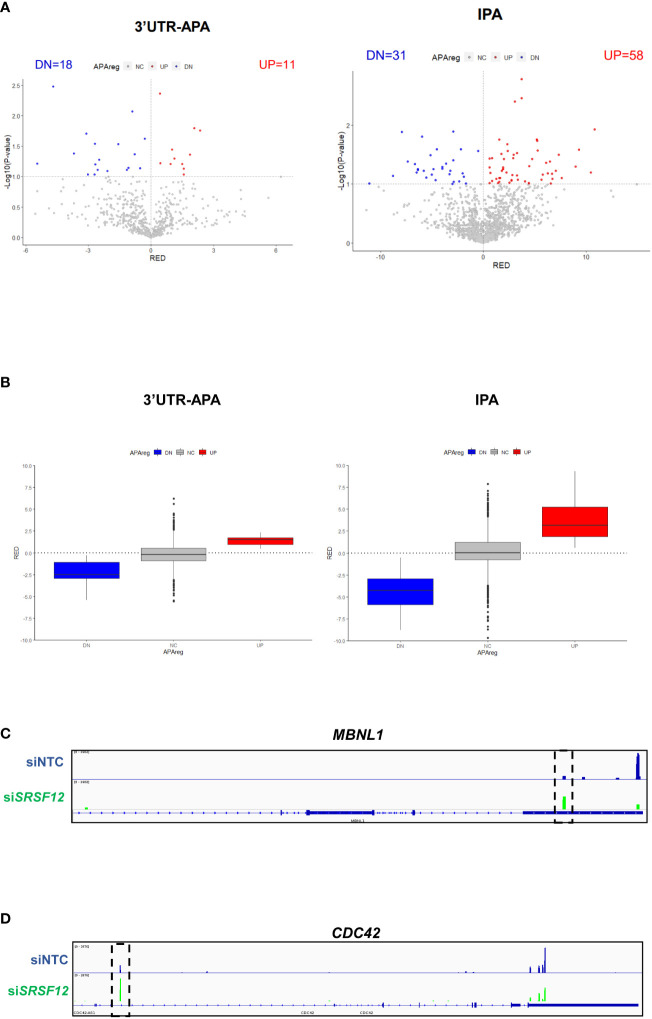
*SRSF12* knockdown induces 3’UTR-APA and IPA changes in M1 macrophages. **(A)** Volcano plots of differential 3’UTR-APA (left) and IPA (right) in si*SRSF12 vs* siNTC in M1 macrophages. Red dots represent genes with Relative Expression Differences (RED) >0, corresponding to upregulation of the lengthened isoform **(A)** or upregulated IPA **(B)**. Blue dots represent genes with RED<0, corresponding to upregulation of the shortened isoform **(A)** or downregulation of IPA **(B)**, and p < 0.1, gray dots represent non-significantly expressed genes. **(B)** Left, box plot with relative expression differences for 3’UTR-APA shortening (DN), 3’UTR lengthening (UP) and non-significant (NC) and right, Upregulated IPA (UP), Downregulated IPA (DN) and non-significant IPA (NC). The APAreg was calculated using a p-value<0.1 based on an unpaired t-test and a RED difference of 5%. **(C)** *MBLN1* IGV gene profile and transcript counts in si*SRSF12* (green) and siNTC (blue) M1 macrophages shows upregulation of the proximal isoform upon SRSF12 knockdown (dashed box). **(D)** *CDC42* IGV gene profile and transcript counts in si*SRSF12* (green) and siNTC (blue) M1 macrophages shows upregulation of the IPA isoform upon SRSF12 knockdown (dashed box).

We next analyzed 3’UTR-APA and IPA events in si*SRSF12 vs* siNTC (non-targeting siRNA control) macrophages and observed that there is an increase in short 3’UTR-APA and IPA mRNA isoforms (**Figure 6A**; **Supplementary Data 3**). Box plot representations of 3’UTR-APA and IPA confirm the Volcano plots results and show that there is higher dispersion of values in IPA than in 3’UTR-APA (**Figure 6B**). Interestingly, *MBNL1*, a splicing regulator (73) that was also shown to modulate alternative polyadenylation (74), show a decrease in the distal PAS usage concomitant with an increase in proximal PAS use in the 3’UTR (**Figure 6C**). *CDC42*, which regulates macrophage chemotaxis and acts as an effector of phagocytosis (75, 76), shows upregulation of an IPA mRNA isoform after *SRSF12* knockdown in macrophages (**Figure 6D**).

## Discussion

Elucidating the gene expression and transcriptomic profile of pro-inflammatory macrophages, key immune cells in the tumor microenvironment, provides new insight into the mechanisms of immune evasion and cancer progression. However, the use of primary human macrophages in transcriptomic studies has been hampered by their highly plastic nature and thus different methodologies for monocyte differentiation and macrophage polarization often result in diverse phenotypic outcomes. The use of monocytic cell lines, on the other hand, has the pitfall of not reproducing what occurs in primary cells. We used a robust and previously validated protocol for differentiation and polarization of primary human monocytes into a pro-inflammatory state and performed indirect co-cultures with two different CRC cell lines. We then performed an unbiased transcriptomic study where we assessed simultaneously gene expression, 3’UTR-APA and IPA events. To our knowledge, this is the first study that identifies 3’UTR-APA and IPA mRNA isoforms after pro-inflammatory polarization of primary human macrophages. Our 3’RNA-Seq data yields the biggest known catalog to date of differential usage of alternative 3’ UTRs and IPAs in primary human macrophages after M1 polarization, CRC stimuli and *SRSF12* knockdown, consisting of 30584 total 3’UTR-APA and IPA events, of which 1161 show significant differences. This data portfolio includes 3’UTR-APA and IPA events in key inflammatory-related genes, increasing the spectrum of known alterations in immune cells after pro-inflammatory polarization and exposure to cancer cells. M1 polarization induces substantial changes in macrophage gene expression (3005 genes out of a total of 19328 detected mRNAs, i.e., 15.5%), which triples the 5% of DEGs previously reported in this population (35). In agreement with a pro-inflammatory polarization, upregulated genes are involved in pro-inflammatory functions, while downregulated genes are related to anti-inflammatory and homeostatic functions.

Although pro-inflammatory macrophages are the main population in the colonic microenvironment at the beginning of the tumorigenic process and before immune evasion (6, 7), it has been suggested that during disease progression, the macrophage profile may change toward a more anti-inflammatory and permissive microenvironment (77). Our results are in agreement with these observations, as after 24 hours of CRC co-culture macrophages present an upregulation of several anti-inflammatory genes. Single-cell analyses have identified TAM signatures in cancer (78). After exposure to CRC cells for 24 hours, we identified our macrophage expression profile as belonging to the inflammatory cytokine-enriched TAMs (*G0S2*, *IL1B*, *IL6*, *S100A8*) and pro-angiogenic TAMs (*HES1*, *IL1B*, *IL8*, *S100A8*, *SERPINB2*, *THBS1*)

We identified a 35 DEG signature in co-cultured macrophages with upregulation of anti-inflammatory and pro-tumoral genes. Importantly, we observed that these results are in agreement with *in vivo* data from CRC patients, such as *LRP5*, *CXCL8* and *EREG*. In particular, *LRP5*, a member of the Wnt pathway (45), which presents the highest increase in its expression in macrophages upon CRC co-culture, is overexpressed in tumor-associated macrophages (TAMs) in CRC patients. These results provide new insight into a function for *LRP5* in the macrophage response to CRC.

Our 3’UTR-APA analyses reveal that M1 polarization induces a strong 3’UTR-APA shortening and that affected genes have important functions in the macrophage response to CRC, including in the Wnt and TGFβ pathways. We identified subsets of physiologically relevant genes that undergo differential 3’UTR-APA, including *IL17RA*, the receptor of the pro-inflammatory cytokine *IL17A* that plays important functions in inflammation and cancer (79), and *TP53RK*, also known as *PRPK*, which codes for p53 protein kinase (80). M1 polarization leads to a decrease in the usage of the most distal PAS and to an increase in the use of the most proximal PAS for *IL17RA* and *TP53RK*, which may affect their function.

Although changes in 3’UTR-APA have been described in CRC (81) and in human macrophages infected with vesicular stomatitis virus (30), our study describes for the first time how CRC exposure modifies the 3’UTR-APA profile of primary human macrophages. Co-culture with two distinct CRC cell lines, HCT-15 and RKO, allowed the identification of a subset of mRNA isoforms that are common to both cell lines. As these cell lines differ in several tumorigenic markers such as APC, K-ras, B-raf, TGFBR2 and MLH1 (38), the identification of these new mRNA isoforms in response to CRC cells may provide insight on the crosstalk and signaling occurring between CRC cells and macrophages.

IPA is defined by the usage of an alternative PAS within an intron, as illustrated by the classical example of the membrane-bound and secreted IgM in B cells (82). It has been shown that IPA is a widespread event (24), particularly in immune cells (25), and that it inactivates tumor suppressor genes in leukemia (26). Here, we found that polarization induces IPA changes in macrophages and that there is a negative correlation between differential gene expression and IPA. We identified IPA events that may generate truncated proteins with other functions, as MAP3K8 and WDR33. MAP3K8 is necessary for activation of the MAPK/ERK pathway in macrophages, being key for producing the pro-inflammatory cytokine TNF-α during immune responses. *MAP3K8* expresses an IPA mRNA isoform at low levels in M0, which is increased by 2-fold upon macrophage M1 polarization. Strikingly, this IPA isoform is even more expressed when macrophages are co-cultured with CRC. As this *MAP3K8* mRNA isoform is annotated and a possible protein is predicted, it is likely that this IPA isoform has implications for the response of macrophages to external stimuli. WDR33 binds directly to the AAUAAA and thus has a key function in the mechanism of pre-mRNA 3’ end processing by defining the PAS (57). We found that M1 polarization leads to an increase in the production of a shorter IPA mRNA isoform, which is further increased upon CRC co-culture. It remains to be investigated the function of these short *MAP3K8* and *WDR33* isoforms in macrophages.

The expression of IPA mRNA isoforms depends on the competition between splicing and polyadenylation as illustrated by production of mRNA isoforms for membrane-bound *vs* secreted immunoglobulin and CT/CGRP mRNA isoforms (82–85). The efficiency of splicing is dictated by SR proteins (86). Here we show that *SRSF12/SRp35* (serine/arginine-rich splicing factor 12), which was initially described as an antagonist of SR proteins (72), is highly upregulated after M1 polarization of primary human macrophages, and that *SRSF12* knockdown leads to a strong downregulation in global gene expression. Additionally, *SRSF12* depletion leads to 3’UTR-APA and IPA changes in a number of genes. For example, MBNL1 is a key splicing regulator that also regulates alternative polyadenylation (79) and proximal PAS usage of the mRNA increases upon *SRSF12* knockdown. In addition, there is an increase in IPA in *CDC42*, a small GTPase that regulates macrophage chemotaxis and phagocytosis (75, 76).

Overall, our findings show that pro-inflammatory polarization and CRC co-culture induces 3’UTR-APA shortening and IPA mRNA isoforms with a function in the macrophage response to external stimuli that may elicit a prompt anti-tumor response.

### Methods details

#### Reagents

Macrophage colony stimulating factor (M-CSF, 300-25, Peprotech), lipopolysaccharide (LPS, L4005, Sigma-Aldrich), interferon-gamma (IFN-γ, 300-02, Peprotech) were prepared as in the manufacturers’ instructions. RPMI 1640 medium, streptomycin, penicillin, trypan blue and heat-inactivated fetal bovine serum (FBS) were obtained from Gibco. Bovine Serum Albumin (BSA) was obtained from NYZTech.

#### Cell lines

RKO and HCT-15 cells, both from American Type Culture Collection (ATCC) were maintained in complete Roswell Park Memorial Institute 1640 medium (RPMI) with GlutaMAX and supplemented with 10% FBS, 100 U/mL penicillin and 100 µg/mL streptomycin antibiotic solution. Cells were kept at 37°C, 5% CO_2_ in a humidified incubator, and subcultured every 3 to 5 days to maintain subconfluency. Trypan blue dye (Gibco) exclusion test was used to assess cell viability.

#### Human monocyte isolation

Briefly, peripheral blood mononuclear cells (PBMCs) were isolated from buffy coats using Ficoll gradient centrifugation, followed by enrichment of CD14^+^ cells using CD14^+^ magnetic beads (Miltenyi Biotech, 130-050-201), according to the manufacturer instructions.

#### Macrophage differentiation and activation

1x10^6^ human CD14^+^ cells were plated on 6-multiwell plates and maintained in RPMI 1640 medium supplemented with 10% FBS (Gibco), 100 U/mL penicillin (Invitrogen), 100 µg/mL streptomycin (Invitrogen) and 50 ng/mL M-CSF (Peprotech), and differentiated for 7 days. On day 7, media was refreshed and cells were polarized using 100 ng/mL LPS (Sigma-Aldrich) and 25 ng/mL IFN-γ (Peprotech) in RPMI 1640 medium supplemented with 2% FBS without antibiotics for additional 24 h. Non-polarized M0 macrophages were maintained in RPMI 2% FBS without antibiotics. At the end of the experiment, spent culture media were collected for ELISA.

#### Macrophage-cancer cell indirect co-cultures

24 hours after macrophage polarization, 3x10^5^ RKO or HCT-15 cells were seeded into transwell inserts with 1.0 µm pore size membrane (Corning 353102) and put on top of 1x10^6^ M1 macrophages. Co-cultures were maintained in complete RPMI 1640 medium for 24 h at 37°C and 5% CO2 in a humidified atmosphere, after which both cell populations and cell culture media were collected for downstream assays. M1 macrophages not exposed to co-culture were maintained in complete RPMI for the duration of the experiment.

#### Macrophage siRNA knockdown

Briefly, 50 nM of a pool of 4 *SRSF12* siRNAs (Dharmacon ONTARGETplus SMARTpool siSRSF12) or 50 nM of a pool of a non-targeting siRNA control (Dharmacon ONTARGETplus SMARTpool siNTC) were transfected into 1.5x10^6^ M1 macrophages in 6-multiwells using 4 µL GenMute for Primary Human Macrophages (SignaGen) as a transfection agent, according to manufacturer’s instructions. Knockdown levels were assessed by RT-qPCR.

#### Immunofluorescence

Macrophages grown on coverslips were fixed using 4% paraformaldehyde in 1X PBS for 15 min. Cells were quenched in 50 mM ammonium chloride for 10 min, permeabilized in 0.2% Triton X-100 in 1x PBS for 5 min and incubated in 5% BSA blocking solution for 30 min, all at room temperature, before incubating for 1 h with mouse anti-ß-tubulin and for 45 min with anti-mouse AlexaFluor 488 secondary antibody, protecting from light. Coverslips were then incubated with Phalloidin-FITC for 15 min at room temperature, and mounted with DAPI-Vectashield. Confocal images were acquired in a Leica Spectral Confocal TCS-SP5 AOBS (Wetzlar, Germany) microscope, using the 40x or 63x oil objectives. All experimental conditions were surveyed using the same microscope conditions. Images were analyzed and processed using the FIJI package for Image J (91).

#### Flow cytometry

Macrophages were incubated with Accutase (eBioscience, Asymetrix) for 30 min at 37°C, harvested by gentle scraping, washed with 1x PBS and resuspended in FACS buffer (1x PBS, 2% FBS, 0.02% sodium azide), blocked with FACS blocking reagent and resuspended in diluted antibodies for 30 min at 4°C in the dark: CD14-FITC (BD 555397, clone M5E2, 1:50 dilution), CD86-APC (Exbio 1A-531, clone BU63, 1:25 dilution), and CD163-PE (BD 556018, clone GHI/61, 1:25 dilution). Unstained cells were used as control. Cell surface markers were detected on FACS Canto II (BD Biosciences), using FACS Diva^®^ software, and data were analyzed using FlowJo software v.10.4.

#### *In silico* analyses

Nucleotide sequences and mRNA profiles were obtained from NCBI and Ensembl genome browsers. The Ensembl and UCSC genome browsers and the APAdb and Aceview databases were accessed to search for alternative polyadenylation sites on the 3’ UTR of human genes.

#### RNA extraction

Cells were washed with ice-cold PBS and resuspended in 500 µL TRIzol (Invitrogen) to extract total RNA according to manufacturer’s instructions, with the exception that all samples were incubated overnight at -80°C during isopropanol precipitation. Total RNA quantity was determined with a Nanodrop 1000 spectrometer (Thermo Fisher Scientific) or a Qubit Fluorometer, using Qubit RNA HS Assay Kit (ThermoFisher).

#### cDNA synthesis and quantitative real-time PCR

Total RNA was digested with DNase I (Roche) for 25 min at 37°C, inactivating the enzyme for 10 min at 80°C. RNA was reverse transcribed using SuperScript IV (ThermoFisher) and random hexamers, according to manufacturer’s instructions. RT-qPCR reactions were performed in triplicate using SYBR Select Master Mix (Applied Biosystems) following the manufacturer’s protocol and 0.125 µM of the primer pairs listed in **Supplementary Table 1**. Negative controls without reverse transcriptase were used to detect possible gDNA contamination. Relative expression was calculated relative to the reference gene *18S*. For quantification of the relative expression RT-qPCR reactions were performed in triplicates using TaqMan Probes and Master Mix (Applied Biosystems). Relative expression was calculated relative to the reference gene *ACTB*. Results were analyzed applying the ΔΔCt method (92).

#### Primer design

For RT-qPCR primers were designed to the coding and most distal PAS (pA2), as previously described (93, 94). Primer sequences used in the RT-qPCRs are listed on **Supplementary Table 1**.

#### Chromatin-bound fractionation

Total RNA from macrophages was fractionated as described in (34). Briefly, 6x10^6^ cells for each condition were washed twice with ice-cold 1x PBS, spun down, and cell pellets were lysed with HLB/NP40 buffer (10 mM Tris-HCl pH 7.5/10 mM NaCl/2.5 mM MgCl_2_/0.05% NP-40 Igepal (Sigma)), carefully underlaid with HLB/NP40/Sucrose (HLB/NP-40 + 25% sucrose) and centrifuged at 208 g for 5 minutes at 4°C to isolate the nuclei from the cytoplasm. The chromatin fraction was isolated *via* treatment with NUN1 solution (20 mM Tris-HCl pH 7.9/75 mM EDTA/50% v/v glycerol/Protease Inhibitor cOmplete 1x (Roche) added fresh) and NUN2 buffer (20 mM HEPES-KOH pH 7.6/300 mM NaCl/0.2 mM EDTA/7.5 mM MgCl_2_/1% v/v Igepal/1 M Urea), with 15 min incubating on ice, followed by centrifugation at 15680 g for 10 minutes at 4°C, separating from the nucleoplasm supernatant. The RNA-bound chromatin pellet was then resuspended in high salt buffer HSB (10 mM Tris-HCl pH 7.5/500 mM NaCl/10 mM MgCl_2_) and digested with TURBO DNase (Invitrogen) and Proteinase K (10 mg/mL; Ambion) for 10 min each at 37°C and 182 g for 10 min. All buffers were ice-cold. RNA was then extracted from the cytoplasmic and chromatin-bound fractions with Trizol. To ensure adequate fraction separation was achieved, *MALAT1* RNA levels were measured by RT-qPCR in cytoplasmic and chromatin-bound fractions; *MALAT1* presence was only detected in the chromatin-bound fraction.

### RNA-Seq

#### Library preparation

Library preparation for 3’ RNASeq from healthy blood donors was performed as (32). Briefly, for M0, M1 and co-cultured macrophage samples, polyA^+^ mRNA was obtained from total RNA using Dynabeads mRNA purification kit (ThermoFisher), according to manufacturer’s protocol. QuantSeq Rev (Lexogen) mRNA libraries were generated following the manufacturer’s protocol. Library size and quantification was performed using Agilent TapeStation and KAPA Library Quantification Kit (KAPA Biosystems). Libraries were sequenced with NextSeq500 using single-end 75-nucleotide reads in the Lexogen Sequencing Facility (Vienna). The protocol for 3’RNA-Seq of si*SRSF12* and siNTC conditions was identical to the protocol used for M0, M1 and co-cultured macrophages, with the exception of being performed using total RNA.

Chromatin-bound RNA-Seq was performed in M1 and co-cultured macrophage as previously described (95). rRNAs were removed using Ribo-Zero Gold rRNA removal Kit (Human/Mouse/Rat) (Illumina) according to the manufacturer’s protocol. Chromatin-bound RNA libraries from three biological replicates were produced using NEBNext Ultra II Directional Prep Kit for Illumina (New England Biolabs) according to the manufacturer’s protocol, and sequenced with Illumina NextSeq High-Output Kit, 75 cycles, using paired-end 42-nucleotide reads.

#### Data processing

In all datasets, quality control was performed using FastQC and adapters were trimmed with Cutadapt (v1.13), discarding reads under 10 bases.

For 3’RNA-Seq, raw sequencing data were filtered to detect accurate poly(A) signals, since this sequencing technique may have intrinsic leakiness in polyadenylation signal discovery. Briefly, the input fastq file was mapped to the hg38 reference and three filters were applied in sequence: 1) a Transcript Termination Site (TTS) filter, removing transcripts not lying within ~10 nt of an annotated transcript end site; 2) a Motif filter removed all sequencing peaks whose read start contained a subset of hexamer sequences upstream and 3) a downstream A content filter checked the amount of As in a window downstream of the read start; if a given peak passes the validated threshold, the read is considered internal priming and is removed. All reads passing the three filters are retained as non-internal priming. Total fragments passing all filters ranged from 47.4-70.7% (**Supplementary Table S2**). Data were aligned to the reference human genome (hg38) using STAR (96). Aligned data had 75.6%-85.4% overall read mapping rate (**Supplementary Table S2**). For *siSRSF12* data, aligned data had 33.6%-43.8% overall read mapping rate (**Supplementary Table S3**).

For ChrRNA-Seq, data were aligned to the reference human genome (hg38) using TopHat (v.2.1) (87), allowing read pairs to be separated by 3 kb and only one alignment to the reference genome for each read. Aligned data had 89%-91.2% overall read mapping rate (**Supplementary Table S4**).

In all datasets, aligned reads were processed with SAMtools (88) and counted with htseq-count (89) and gene expression differences were calculated using DESeq2 (90). Sequencing read distribution through the genome was visualized in IGV, v7.0, using BedGraphToBigWig.

To increase statistical robustness to our analysis, we only considered genes with >5 mean TPM, Log2Fold Changes ≥ 1 or ≤ -1, and adjusted p-value < 0.05, calculated by DESeq2 (97). Additionally, to establish data set reproducibility, we used, used 3 independent experiments with macrophages from three donors and analyzed the Pearson correlation values. R values exceeded 0.9 for each condition, exceeded 0.8 between pro-inflammatory and co-cultured macrophages, and exceeded 0.5 between M0 and M1 (**Figure S2D**).

mRNA isoform expression differences were calculated in the 3’RNA-Seq data using APAlyzer to generate lists of genes showing differential expression of alternative mRNA isoforms by alternative polyadenylation. APAlyzer is a Bioconductor package which analyzes 3’UTR-APA and IPA based on annotated, conserved polyA sites at the recommended cutoff of relative expression between conditions (5%), using p-value<0.05 with the exception of siSRSF12 3’RNA-Seq data where p<0.1. For downstream analyses, we focused on isoforms of protein-coding genes as per ENSEMBL databases, excluding out-of-scope isoforms such as NMD.

3’UTR-APA relative expression difference (RED) was determined by Log2(aUTR read number/cUTR read number) where cUTR is the region between the stop codon and the first PAS and aUTR is the region between the first and the last PASs. To calculate IPA relative expression log2((RDIU - RDID)/RDTE) was used, where RDIU is the read density of the intronic upstream region of the IPA site, RDID is the read density of the intronic downstream region of the IPA site, and RDTE is the read density of the constitutive region in the 3’terminal exons. PASs were localized using the polyA_DB for the hg38 version. The relative expression difference (RED) was calculated as the difference in log2(RPM ratio) between two groups.

To clarify the identity of each mRNA isoform being produced in each condition, mRNA isoforms detected by 3’RNA-Seq were compared to Chr-bound RNA-Seq data from M1 and M1 + RKO macrophages and to transcripts deposited in the ENSEMBL database.

For *SRSF12* knockdown 3’RNA-Seq, the bioinformatic pipeline was the same, with the exception that the initial filtering algorithm was not applied.

### Candidate gene selection

Criteria for 3’RNA-Seq candidate gene validation were as follows: DGE candidates were selected for large fold change differences between control and co-cultured macrophages, robustness of response after macrophage co-culture with both CRC cell lines, and relevance to macrophage biology. mRNA isoform candidates were selected according to the following criteria: i) prevalent 3’ UTR mRNA isoform shift between the 3’ RNA-Seq signal in the two mRNA isoforms with the highest reads, ii) robustness of response after macrophage polarization and co-culture with both cell lines (increase in proximal or distal mRNA isoform) and iii) relevance to macrophage biology and inflammation.

### TCGA analysis of patient survival

Datasets for colon carcinoma and normal tissue with CD68^high^ expression were retrieved from the Cancer Genome Atlas [TCGA (98)] and analyzed for expression levels. The expression of genes was quantified as FPKM (fragments per kilobase million), provided by TCGA consortium. The normal tissue median values were used as threshold to define “high” and “low” expression levels for each identified DEGs in cancer patients, quantified as FPKM (fragments per Kilobase million). The correlation index (R) indicated a moderate correlation if between 0.25 and 0.35, and a strong correlation if higher than 0.35.

### Gene ontology and gene set enrichment analysis

Gene Ontology (GO) enrichment analysis was performed on the genes showing APA differences, independently of its overall gene expression values, using PANTHER v. 16 (http://www.pantherdb.org/) focusing on the Molecular Function. Background for GO analyses on PANTHER was the human genome, Reference Proteome 2021_03. Results from all gene sets used were compiled into tables and ordered by p-value. Terms with non-significant p-values were removed from the analysis and the remaining GO terms were ordered by number of input genes. GO entries with general terms (e.g., “regulation of biological process”) were removed and the top terms were presented in a graph.

On genes with differential expression, Gene Set Enrichment Analysis was performed using GSEA software (Broad Institute). Briefly, three tables were constructed containing the results of all DEGs analyses, one for each comparison (M1 *vs*. M0; M1 + RKO *vs*. M1, M1 + HCT-15 *vs*. M1). Tables were ordered by log2(fold-change), converted into text files saved as.rnk format, and uploaded into the GSEA software. In the option “Run GSEA”, each expression dataset was loaded into the respective field and analyzed with the following gene sets: Hallmark gene sets, Canonical pathways (BioCarta, KEGG, PID, Reactome), Regulatory target gene sets (Transcription factor), Ontology gene sets (GO Biological Process, GO Cellular Component, GO Molecular Function), Oncogenic signature gene sets, Immunological signature gene sets (ImmuneSigDB). Other options selected included classic Enrichment statistic, increasing the maximum size to 5000 and decreasing the minimum size to 1, in order to make the broadest possible analysis. Results from all gene sets used were compiled into a table and ordered by p-value. GO terms with not significant p-values were removed from the analysis and the remaining GO terms were ordered by number of input genes. Entries with general terms (e.g., “regulation of biological process”) were removed. The resulting top GO terms were plotted.

### Enzyme-linked immunosorbent assay

Cytokines and metabolites were quantified in the culture media of mono and co-cultures by ELISA using kits from ThermoFisher and BioLegend, according to manufacturer’s instructions.

### Western blot

Macrophage lysates were prepared with RIPA buffer (50 mM Tris-HCl pH 7.5/1% NonidetP (NP)-40/150 mM NaCl/2 mM EDTA) with protease inhibitors (Protease Inhibitor cOmplete 1x). Protein concentration was determined through the Bradford method and 25 µg of protein was loaded and run in a 7.5% (for LRP5) or 10% acrylamide gel, which was subsequently transferred into nitrocellulose membranes (GE Healthcare), blocked with 5% non-fat powdered milk or BSA in TBS + 0.1% Tween 20 (TBS-T) for 1 hour, and incubated overnight with primary antibodies at 4°C (**Supplementary Table S5**). HRP-conjugated secondary antibodies (SCBT) were incubated for 1 hour at RT. Signal was detected by incubation with ECL Substrate (GE Healthcare), using the Chemidoc Imaging System (BioRad) and band intensity was measured in the ImageLab Software (BioRad).

### Statistical analysis

All presented data are mean ± standard deviation (SD). Data were evaluated for normal distribution through the Kolmogorov-Smirnov test. Comparisons between two independent groups were performed using the paired Student’s t-test or the Wilcoxon test. Except where otherwise stated, p-values <0.05 were considered statistically significant (* = p < 0.05; ** = p < 0.01; *** = p < 0.005; **** = p < 0.0001). Graphs were made in the GraphPad Prism software, using version 7.0a for Mac, GraphPad Software, La Jolla California USA, www.graphpad.com.

## Data availability statement

The data discussed in this publication is deposited in NCBI’s Gene Expression Omnibus and are accessible through GEO Series accession number GSE163726.

## Ethics statement

The studies involving human participants were reviewed and approved by Centro Hospitalar Universitário Sao Joao Ethics Committee, protocol 90/19. The patients/participants provided their written informed consent to participate in this study.

## Author contributions

JW and IPC performed all molecular biology and transcriptomic experiments, JF performed some FACS analyses, AMC performed TCGA, TN performed the RNA library preparation. JW, FLM and MT performed all bioinformatics analyses. JW, MJO, AM, SM and NJP designed the project and wrote the paper.

## Supplementary material

Supplementary Figure 1 **(A–E)** Inflammatory profile characterization of M1 normalized to M0. **(A–C)** Percentage of positive cells for CD14 (**A**, n=6), CD86 (**B**, n=6) and CD163 (**C**, n=4) **(D)**. Pro-inflammatory marker *CCR7* and anti-inflammatory marker *TGFB* measured by RT-qPCR (n=3). **(E)** IL1ß secretion in M1 *vs*. M0 macrophages, measured by ELISA (n=3). A-D Student’s t-test: * = p < 0,05; ** = p < 0,01. M0 in black, M1 in blue, M2 in light gray. **(F)**. GSEA terms of genes upregulated (top) and downregulated (bottom) in M1 *vs*. M0. **** = p < 0,0001.

Supplementary Figure 2 **(A)** Confocal image of macrophages co-cultured with CRC cell line HCT-15 for 24 hours as shown in (M1 + HCT-15). DNA stained in blue, actin stained in red, tubulin stained in green. **(B, C)**. Inflammatory profile characterization of M1 + RKO *vs*. M1. Percentage of positive cells (left) and median fluorescence intensity (right) for CD14 (B), and CD86 (C) measured by flow cytometry in M1+RKO (red) *vs*. M1 (blue). **(D)** Heatmap of Pearson correlation coefficients for 3’ RNA-Seq samples, using transcript counts per million (CPM); Scale: 0-1, 0 = no correlation between populations; 1= total correlation between populations (n=3). **(E)** Principal Component Analysis (PCA) of M0, M1 and co-cultured macrophages data. The plot displays the first two principal components, with the percentage of explained variance indicated on the axes. The colors represent the different groups. **(F)** RT-qPCR validation of common 3’RNA-Seq DEGs in co-cultured macrophages. Top: upregulated genes *SEMA6B* (n=3*)*, *THBS1* (n=4), Bottom: downregulated genes *RGS3* (n=4) and *P2RY13* (n=3). p-value calculated using Student’s t-test * = p < 0,05; ** = p < 0,01. **(G)** Representative donor heatmap of genes exclusively upregulated in M1 + RKO *vs*. M1 (left) M1 + HCT-15 *vs.* M1 (right), ordered by fold change (n=3, p-value calculated using DESeq2 test through the Benjamini-Hochberg method. * = p < 0,05; ** = p < 0,01; *** = p < 0,005; **** = p<0,0001). **(H)** Venn diagram showing the common genes differentially expressed in M0 vs M1 and M1 vs M1 co-cultures with RKO and HCT15 datasets.

Supplementary Data Sheet 1 Fold change and p-value of genes with differential expression calculated by DESeq2 in M1 *vs*. M0 macrophages, M1+RKO *vs*. M1 and M+HCT15 *vs*. M1.

Supplementary Data Sheet 2 Relative expression differences and p-value of genes with APA and IPA differences calculated by Apalyzer in M1 *vs*. M0 macrophages, M1+RKO *vs*. M1 and M+HCT15 *vs*. M1.

Supplementary Data Sheet 3 Fold change and p-value of genes with differential expression calculated by DESeq2, 3’UTR-APA and IPA calculated by Apalyzer, in M1 si*SRSF12 vs*. M1 siNTC macrophages.
