# Supplementary material for: Pro-inflammatory polarization and colorectal cancer modulate alternative and intronic polyadenylation in primary human macrophages
Source: Front Immunol. 2023 Jun 8;14:1182525. doi: 10.3389/fimmu.2023.1182525 (PMC10286830; doi:10.3389/fimmu.2023.1182525)
Supplement: Supplementary Figure 1 — (A–E) Inflammatory profile characterization of M1 normalized to M0. (A–C) Percentage of positive cells for CD14 (A, n=6), CD86 (B, n=6) and CD163 (C, n=4) (D). Pro-inflammatory marker CCR7 and anti-inflammatory marker TGFB measured by RT-qPCR (n=3). (E) IL1ß secretion in M1 vs. M0 macrophages, measured by ELISA (n=3). A-D Student’s t-test: * = p < 0,05; ** = p < 0,01. M0 in black, M1 in blue, M2 in light gray. (F). GSEA terms of genes upregulated (top) and downregulated (bottom) in M1 vs. M0. **** = p < 0,0001. [file DataSheet_1.pdf]

Pro-inflammatory polarization and colorectal cancer modulate alternative and intronic polyadenylation in primary human macrophages

Wilton *et al.*

Supplementary figures

Supplementary Figure S1

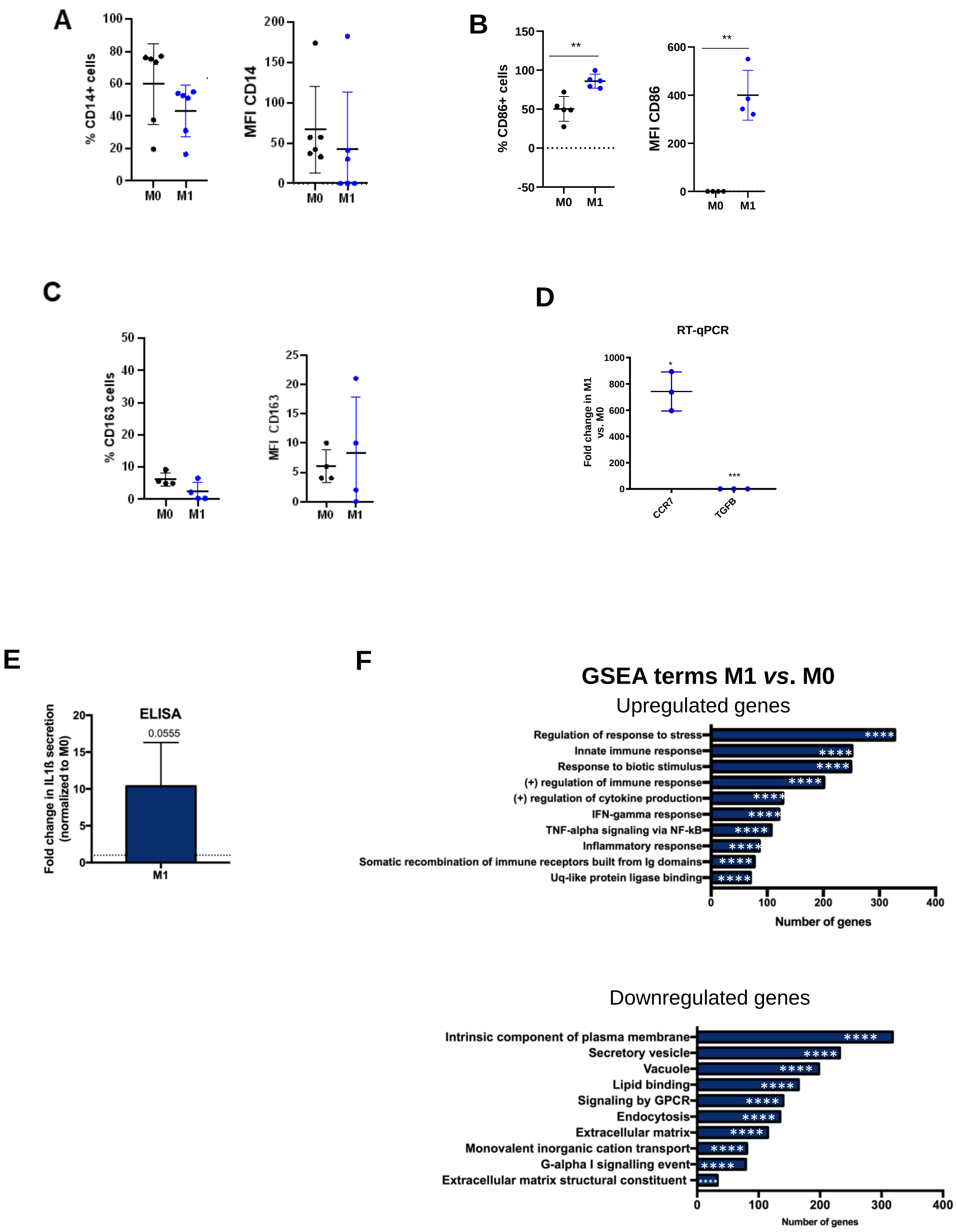

Supplementary Figure S2

A

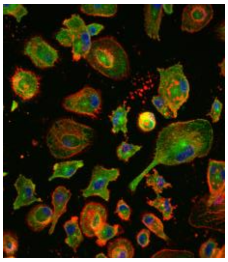

M1 + HCT-15

B

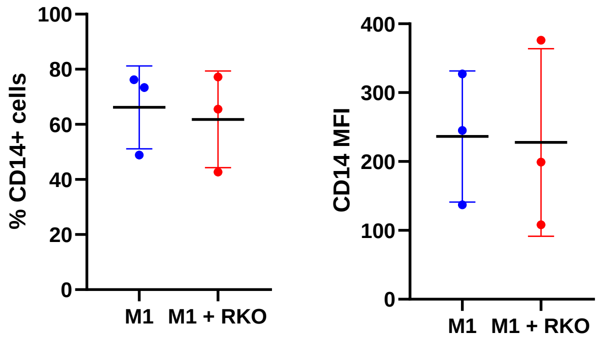

C

FACS

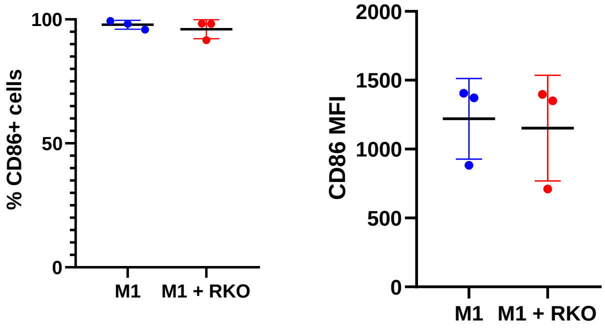

D

3' RNA-Seq – Pearson correlation

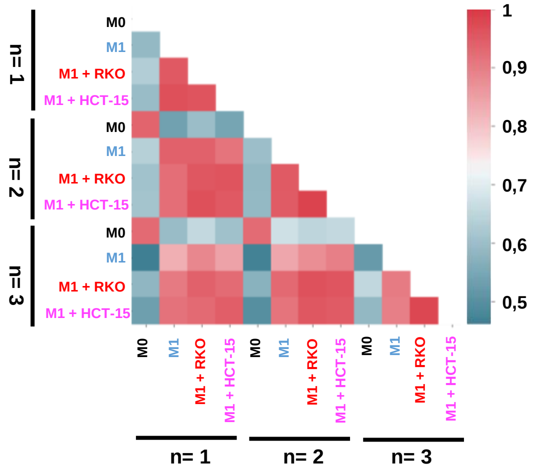

Supplementary Figure S2

E

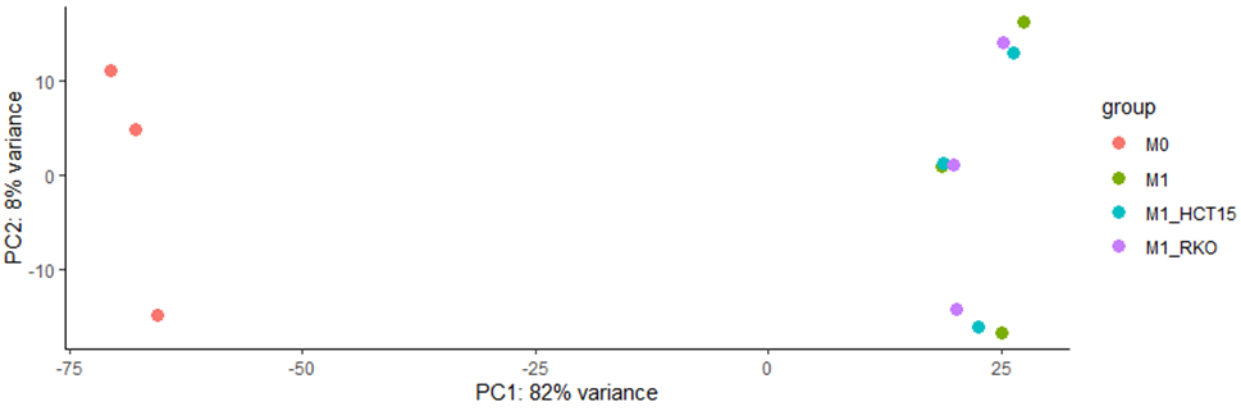

F

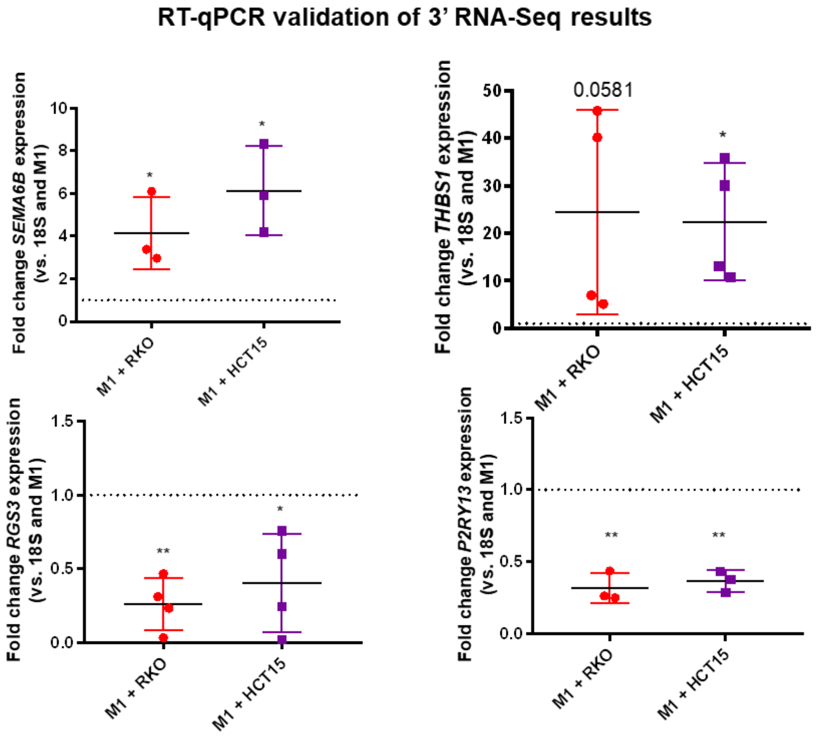

Supplementary Figure S2

G

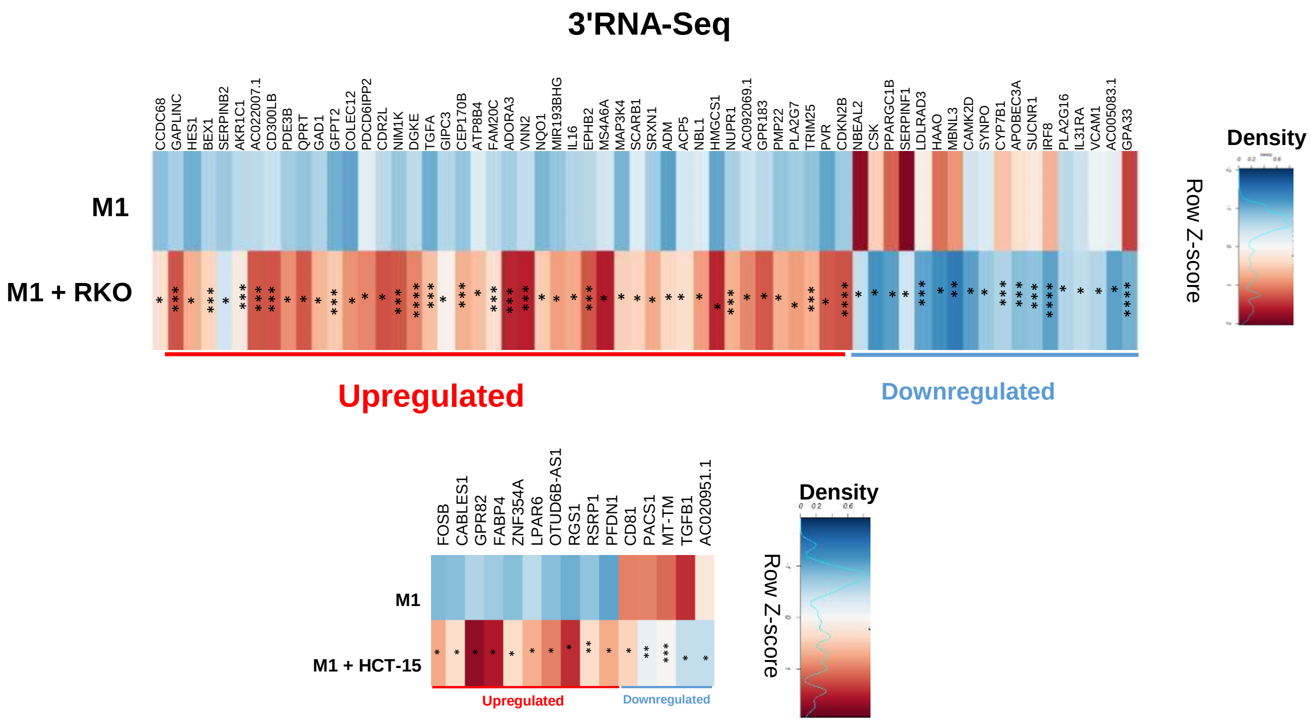

H

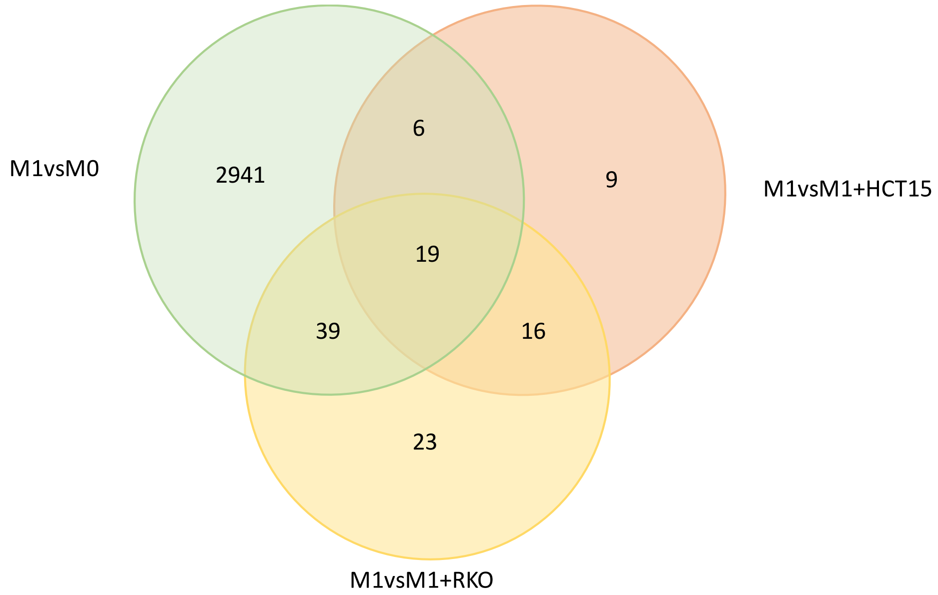

Supplementary Figure S3

A

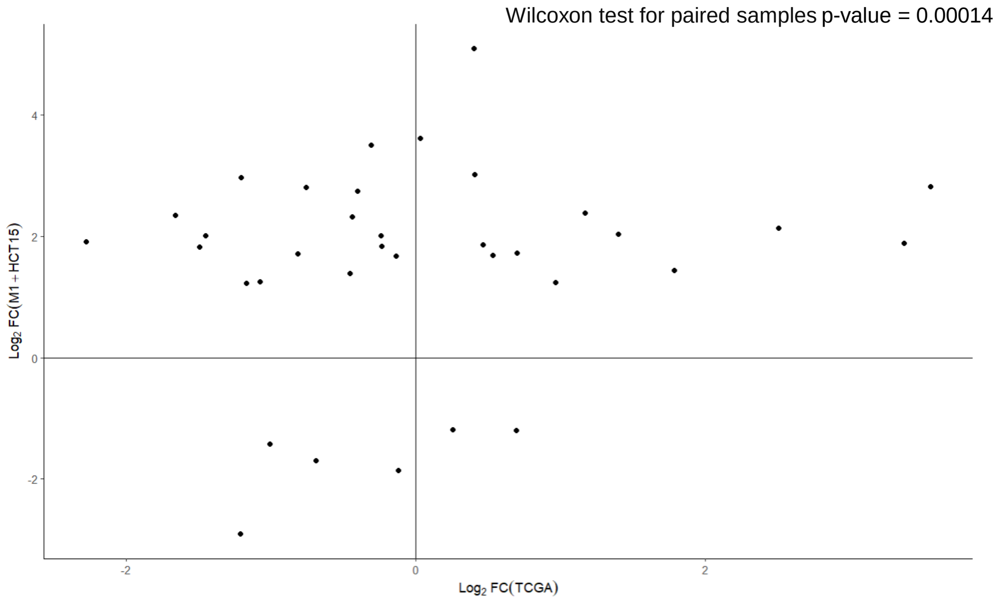

B

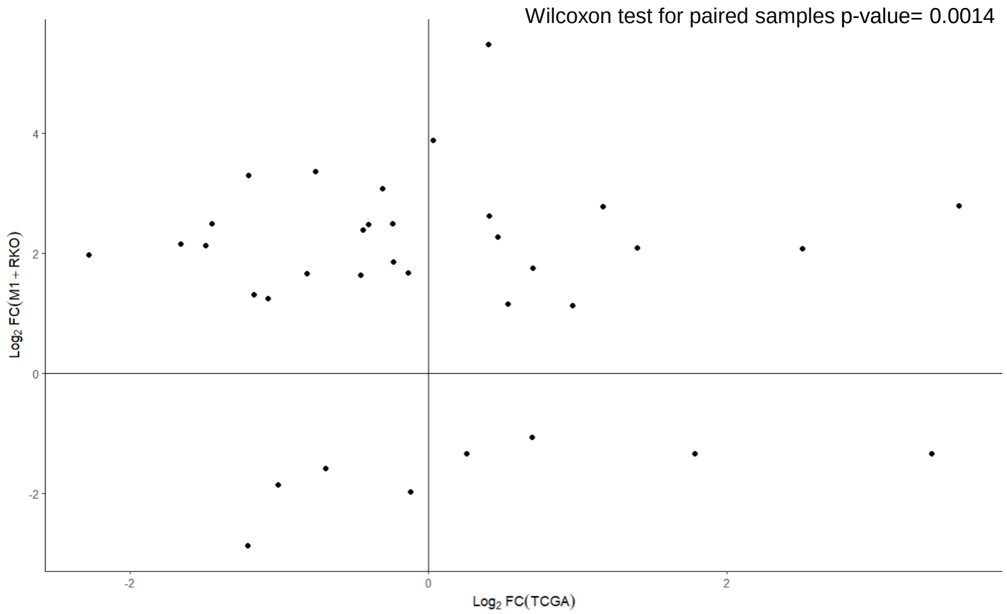

Supplementary Table S1- Primer pairs used for RT-qPCR validation.

Primer name	5'-3' sequence
*LRP5*_fwd	AACATCAAGCGAGCCAAGGA
*LRP5*_rev	CGGCTGTAGATGTCGATGCT
*SEMA6B*_fwd	TACTCGGGGTGTATGAAGAACTG
*SEMA6B*_rev	GGACACGTCCTGCTCAAAGG
*THBS1*_fwd	AGTCGTCTGCAACAACCC
*THBS1*_rev	ACAGGCATCCATCAATTGGACA
*RGS3*_fwd	CAGGCATGCAAGGAGGTCAA
*RGS3*_rev	GCGAGGGTACGAGTCCTTTT
*P2RY13*_fwd	TCCCAAAGGTGACACTGGAAG
*P2RY13*_rev	GGATGCCGGTCAAGAAAACC

Supplementary Table S2 – Total reads, percentage of reads passing filters after pre-processing, respective input read and uniquely mapped reads in 3' RNA-Seq of M0, M1 and co-cultured macrophages data.

Libraries	Sample	Total reads	Pre-processed reads passing filter (%)	Input reads	% Uniquely mapped reads
1	n=1 M0	51830315	65.218	28,035,543	83,1
2	n=1 M1	43059588	69.5065	25,491,051	75,6
3	n=1 Mac + RKO	52246262	66.0313	31,733,385	77,9
4	n=1 Mac + HCT15	42146977	68.1757	25,414,996	77,9
5	n=2 M0	58612102	50.0446	21,175,635	85,4
6	n=2 M1	45231704	70.3475	27,450,875	76
7	n=2 Mac + RKO	46246987	65.8951	28,013,142	78,5
8	n=2 Mac + HCT15	50739567	65.7408	34,958,610	77,4
9	n=3 M0	45196922	59.2683	22,766,165	83,6
10	n=3 M1	57164722	47.4429	25,282,986	78,3
11	n=3 Mac + RKO	49798508	59.8334	27,686,265	77,5
12	n=3 Mac + HCT15	51151485	64.9537	29,334,724	76,8

Supplementary Table S3 – Total reads, uniquely mapping reads and percentage of uniquely mapped reads in 3’ RNA-Seq of siNTC and *siSRSF12*.

Libraries	Sample	Total reads	Uniquely mapped reads	% Uniquely mapped reads
1	n=1 siNTC	55047153	23656401	42,97
2	n=1 siSRSF12	46738604	15802926	33,81
3	n=2 siNTC	59183648	25938268	43,83
4	n=2 siSRSF12	46388770	15578915	33,58

Supplementary Table S4 - Percentage of reads passing filters after pre-processing, respective input read and uniquely mapped reads in ChrRNA-Seq data.

Libraries	Sample	Input reads	Uniquely mapped reads	% of mapping pairs
1	n=4 M1	132,282,932	118,810,242	89,8
2	n=4 M1 + RKO	156,488,832	142,596,491	91,1
3	n=5 M1	147,426,396	131,189,998	89
4	n=5 M1 + RKO	120,061,720	109,392,734	91,1
5	n=6 M1	196,644,824	176,867,389	89,9
6	n=6 M1 + RKO	167,811,172	153,049,138	91,2

Supplementary Table S5: List of reagents and resources.

Reagent or Resource	Source	Identifier
Mouse monoclonal CD14-FITC (clone M5E2, used for flow cytometry)	BD	Cat# 555397; RRID: AB_395798
Mouse monoclonal CD86-APC (clone BU63, used for flow cytometry)	Exbio	Cat# 1A-531-T100; RRID: AB_10734084
Mouse monoclonal CD163-PE (clone GHI/61, used for flow cytometry)	BD	Cat# 556018; RRID: AB_396296
DAPI-Vectashield (for immunofluorescence)	Vector Laboratories	Cat H-1200-10
Mouse monoclonal anti-α-tubulin	Sigma-Aldrich	Cat# T9026
Anti-mouse AlexaFluor 488 conjugated secondary antibody	Life Technologies	Cat # A32723
Alexa Fluor 647 Phalloidin	Life Technologies	Cat # A22287
Mouse anti-hsc70	SCBT	sc-7298
Rabbit anti-LRP5	Cell Signaling	5731S
Rabbit anti-MAP3K8	Abcam	ab137589
TURBO DNase	ThermoFisher Scientific	Cat# AM2238
M-CSF	Peprotech	Cat# 300-25
LPS	Sigma-Aldrich	Cat# L4005
IFN-γ	Peprotech	Cat# 300-02
NEBNext Ultra II Directional RNA Library Prep Kit for Illumina	NEB	Cat# E7760
NextSeq High-Output v2 Kit, 75 cycles	Illumina	Cat# FC-404-2005
Superscript IV Reverse Transcriptase	ThermoFisher	Cat# 18090050
Ribo-Zero Gold rRNA removal kit (Human/Mouse/Rat)	Illumina	Cat# MRZG12324
QuantSeq 3'mRNA-Seq library prep kit REV for Illumina	Lexogen	Cat# 016.24
QuantSeq 3'mRNA-Seq PCR Add-on Kit for Illumina	Lexogen	Cat # 020.96
Sybr Green I	ThermoFisher	Cat # S7563
ELISA Kits	Biolegend<br>ThermoFisher<br>Abcam	Cat # 430504 (Human IL-12p70)<br>Cat # 88-7261-88 (Human IL-1ß)<br>ab213775 (Human epiregulin), ab193716 (Human thrombospondin)
**Software and Algorithms**	**URL**	
GENCODE release 38	https://www.gencodegenes.org/	
FastQC	http://www.bioinformatics.babraham.ac.uk/projects/fastqc/	
Cutadapt (v.1.13)	https://cutadapt.readthedocs.io/en/stable/installation.html	
Bowtie2 (v.2)	http://bowtie-bio.sourceforge.net/bowtie2/index.shtml	
Tophat (v. 2.0.13)	https://ccb.jhu.edu/software/tophat/index.shtml	
STAR	https://github.com/alexdobin/STAR	

Samtools (v. 1.6)	http://www.htslib.org/doc/samtools.html
htseq-count	https://htseq.readthedocs.io/en/release_0.11.1/count.html
DESeq2	http://bioconductor.org/packages/devel/bioc/vignettes/DESeq2/inst/doc/DESeq2.html
APAlyzer	https://bioconductor.org/packages/release/bioc/html/APAlyzer.html
Ggplot2	https://ggplot2.tidyverse.org/
Heatmap	R Studio, v. 3.4.2
Deeptools2	https://deeptools.readthedocs.io/en/develop/
Seaborn library	Python, v.2.7
